# How Sequentially Changing Reward Prospect Modulates Meta-control: Increasing Reward Prospect Promotes Cognitive Flexibility

**DOI:** 10.3758/s13415-020-00825-1

**Published:** 2020-09-09

**Authors:** Kerstin Fröber, Gesine Dreisbach

**Affiliations:** Department of Psychology, University of Regensburg, Universitätsstr. 31, D-93053 Regensburg, Germany

**Keywords:** Meta-control, Reward, Flexibility, Stability, Task switching

## Abstract

Meta-control is necessary to regulate the balance between cognitive stability and flexibility. Evidence from (voluntary) task switching studies suggests performance-contingent reward as one modulating factor. Depending on the immediate reward history, reward prospect seems to promote either cognitive stability or flexibility: Increasing reward prospect reduced switch costs and increased the voluntary switch rate, suggesting increased cognitive flexibility. In contrast, remaining high reward prospect increased switch costs and reduced the voluntary switch rate, suggesting increased cognitive stability. Recently we suggested that increasing reward prospect serves as a meta-control signal toward cognitive flexibility by lowering the updating threshold in working memory. However, in task switching paradigms with two tasks only, this could alternatively be explained by facilitated switching to the other of two tasks. To address this issue, a series of task switching experiments with uncued task switching between three univalent tasks was conducted. Results showed a reduction in reaction time (RT) switch costs to a nonsignificant difference and a high voluntary switch rate when reward prospect increased, whereas repetition RTs were faster, switch RTs slower, and voluntary switch rate was reduced when reward prospect remained high. That is, increasing reward prospect put participants in a state of equal readiness to respond to any target stimulus—be it a task repetition or a switch to one of the other two tasks. The study thus provides further evidence for the assumption that increasing reward prospect serves as a meta-control signal to increase cognitive flexibility, presumably by lowering the updating threshold in working memory.

**Electronic supplementary material:**

The online version of this article (10.3758/s13415-020-00825-1) contains supplementary material, which is available to authorized users.

## How sequentially changing reward prospect modulates cognitive flexibility and stability

Prominent theories suggest that cognitive control is best characterized not as a unitary function, but instead as a set of complementary control functions supposedly mediated by differential activity modes of the neurotransmitters dopamine and/or norepinephrine (Aston-Jones & Cohen, 2005; Braver, 2012; Braver et al., 2014; Cohen, Aston-Jones, & Gilzenrat, 2004; Cools & D'Esposito, 2011; Durstewitz & Seamans, 2008; Goschke, 2003, 2013; Hommel, 2015; Miyake et al., 2000). A commonality of these theories is that cognitive control as the basis of goal-directed action is challenged with antagonistic requirements in a constantly changing environment. For example, the control dilemma theory (Goschke, 2003, 2013) emphasizes that adaptive, goal-directed action needs, on the one hand, the ability to maintain goals over time and to shield them against distraction (cognitive stability). On the other hand, it needs the ability to flexibly update goals whenever significant changes in the environment occur (cognitive flexibility). An important question raised by this kind of theories is how control is controlled itself in accordance with a given situation (meta-control, see also Hommel, 2015). That is, how does our cognitive system know when to be stable and when to be flexible? The importance of understanding these meta-control processes is exemplified in psychological disorders that are characterized by a dysregulation of the stability-flexibility balance (for a review see Goschke, 2014): dysregulated, extreme flexibility can result in incoherent and overly distractible behavior like seen in ADHD, whereas extreme stability can result in overly rigid behavior as seen in obsessive-compulsive disorder. Consequently, an important research question in cognitive psychology is to identify the factors that enable a dynamic regulation of meta-control parameters in a context-sensitive manner.

Research so far has identified affect and reward as two influential modulators of the stability-flexibility balance (for reviews see Chiew & Braver, 2011; Dreisbach & Fischer, 2012; Dreisbach & Fröber, 2019; Goschke & Bolte, 2014; Hommel, 2015). While positive affect is typically associated with increased flexibility and reduced stability (e.g., Dreisbach, 2006; Dreisbach & Goschke, 2004; Fröber & Dreisbach, 2012; Hefer & Dreisbach, 2020b), reward usually increases stability (e.g., Fischer, Fröber, & Dreisbach, 2018; Hefer & Dreisbach, 2017, 2020a; Müller et al., 2007). Research from the last decade demonstrates, however, two exceptions from this stabilizing effect of reward: First, only the prospect of performance-contingent reward increases stability, whereas the prospect of non-contingent reward increases flexibility (Fröber & Dreisbach, 2014, 2016a). Note the emphasis on reward *prospect* here (i.e., announcing the opportunity of a reward *before* a reward-eligible performance) because mere reward *reception* (i.e., learning about a reward only *after* a reward-eligible performance) can in fact have different effects (Calcott, van Steenbergen & Dreisbach, 2020; Notebaert & Braem, 2016). Second, in a context with randomly changing reward magnitudes only repeated high reward prospect increases stability, whereas an increase in reward prospect increases flexibility (Fröber & Dreisbach, 2016b; Fröber, Pfister, & Dreisbach, 2019; Fröber, Pittino, & Dreisbach, 2020; Fröber, Raith, & Dreisbach, 2018; Kleinsorge & Rinkenauer, 2012; Shen & Chun, 2011). This suggests that reward prospect can promote either cognitive stability or flexibility depending on performance contingency and the immediate reward history.

The sequential reward effect (increased flexibility when reward prospect increases vs. increased stability when reward prospect remains high) has been demonstrated with two dependent measures of cognitive flexibility, namely switch costs and the voluntary switch rate: It has been shown that an increase in reward prospect from one trial to the next reduced switch costs by accelerating switch reaction times (RTs) and slowing repetition RTs (Shen & Chun, 2011; see also Kleinsorge & Rinkenauer, 2012). Furthermore, Fröber and colleagues used the voluntary task switching paradigm (first introduced by Arrington & Logan, 2004), where participants are free to choose a task repetition or switch on a given trial, and repeatedly found increased voluntary switch rates when reward prospect increased and lowest switch rates when reward prospect remained high (Fröber et al., 2018; Fröber et al., 2019; Fröber & Dreisbach, 2016b).

In a recent review (Dreisbach & Fröber, 2019), we suggested that this sequential reward effect is based on a modulation of the meta-control parameter updating threshold that regulates the balance between stable maintenance and flexible updating of goal representations in working memory (Goschke, 2013; Goschke & Bolte, 2014). Regarding the underlying neurobiological mechanisms, computational neuroscience models suggest that the updating threshold can be understood as attractor states of varying depth (corresponding to working memory representations) in a neural network landscape in the prefrontal cortex (Durstewitz & Seamans, 2008; Rolls, 2010). An attractor state results from a recurrent activation pattern of a neuronal network with excitatory interconnections. Deep attractor states correspond to strong representations that are resistant against interference and hard to switch away from. That is, they are characterized by a high updating threshold and high cognitive stability. Conversely, shallow attractor states are less stable and facilitate switching between different states. That is, the updating threshold is low and cognitive flexibility is high. This depth of attractor states is assumed to be regulated by an interplay of gamma-aminobutyric acid (GABA) and glutamate together with the neurotransmitter dopamine (DA; Durstewitz & Seamans, 2008; Rolls, 2010). More precisely, a DA D1-receptor dominated state is assumed to mediate stability while a DA D2-receptor dominated state mediates flexibility (see also Cools & D'Esposito, 2011, and Cools, 2016, for a similar distinction between diverging modes of DA activity).

Such computational neuroscience models have proven very useful to understand maladaptive dysregulations of the stability-flexibility balance related with psychiatric disorders (for a review see Goschke, 2014). For example, cognitive symptoms of schizophrenia like distractibility have been attributed to diminished stability of representations in prefrontal cortex networks due to diminished D1 receptor efficacy (Rolls, Loh, Deco, & Winterer, 2008b). At the other extreme, symptoms of obsessive-compulsive disorder are suggested to be based on an increased depth of attractor states, which makes each state too stable so that the cognitive system gets stuck (Rolls, Loh, & Deco, 2008a). With respect to the dynamic, context-sensitive regulation of the stability-flexibility balance found in healthy humans, we suggest that increasing reward prospect might work as a signal to lower the updating threshold in working memory, thereby easing the access of any information to working memory. This would result in a state of equal readiness to respond to either a task repetition or switch, that is, a state of cognitive flexibility in general. Conversely, remaining high reward prospect might increase the updating threshold, thereby shielding the just executed task in working memory and rendering task switching more difficult. However, it is not necessary to assume that an increase in reward prospect triggers an increase in cognitive flexibility in general.

## The present study

Because all previous studies (Fröber et al., 2018; Fröber et al., 2019; Fröber et al., 2020; Fröber & Dreisbach, 2016b; Jurczyk, Fröber, & Dreisbach, 2019; Kleinsorge & Rinkenauer, 2012; Shen & Chun, 2011) used task switching paradigms with two tasks only, the reduction in switch costs as well as the increase in voluntary switch rate could also be explained by a less general form of flexibility. Namely, increasing reward prospect could just have facilitated switching to the other of two tasks. That is, it could be a sign of task-specific flexibility, restricted to the two task sets one has to switch between. To investigate whether an increase in reward prospect in fact results in equal readiness to perform any potential task—that is, if it promotes a more generic form of cognitive flexibility—we used a task switching paradigm with three uncued univalent tasks in the present study. Using three univalent tasks instead of two prevents advance preparation for a specific alternative task in case of a task switch (see Chiu & Egner, 2017, Experiment 3, for a similar argument). Furthermore, we assume that having three tasks in random succession makes it very unlikely that participants would try to keep all three tasks active in working memory in order to be prepared especially given the absence of any advance information. If the immediate reward history indeed modulates the stability-flexibility balance by adjusting the updating threshold in working memory, we should still find reduced switch costs under increasing reward prospect but facilitated task repetitions and large switch costs when reward prospect remains high. This would support the hypothesis of sequential changes in reward expectation as a modulator of meta-control processes.

To investigate sequential changes in reward prospect, one has to manipulate two different reward conditions in random succession. A low reward condition is preferable to a no reward condition, because no reward trials have been shown to motivate some participants to completely disengage from the task in these trials (Shen & Chun, 2011). Furthermore, it is important to ensure that performance-contingent reward is tied to a challenging performance criterion, because it can otherwise be perceived as non-contingent reward. As we have outlined above, reward that is perceived as noncontingent or easy gain can have the opposite effect (Fröber & Dreisbach, 2014; Müller et al., 2007). Thus, to prevent disengagement in low reward trials and to assure a motivational effect in high reward trials, low reward trials in the sequential reward paradigm usually require a correct response while high reward trials require a correct and especially fast response for reward receipt (Fröber & Dreisbach, 2016b; Shen & Chun, 2011). Admittedly, this means that low and high reward conditions differ not only with respect to reward prospect, but also with respect to response requirements. This confound, however, is inevitable if the concept of performance-contingent reward is taken seriously. Therefore, we decided to keep the low and high reward manipulation with different response criteria—correct responses for a low reward, correct and especially fast responses for a high reward—for the present study, because it is considered the best way to manipulate performance-contingent reward. To foreshadow, we provide an empirical approach to address this issue in Experiment 3.

To test whether the sequential reward effect on RT switch costs as first demonstrated by Shen and Chun (2011) is still found in a paradigm with three univalent tasks, we conducted two experiments (Experiments 1 and 2) using a forced-choice voluntary task switching paradigm. Reduced switch costs in such a paradigm would be suggestive of a more generic form of cognitive flexibility, because it does not allow advance preparation for a specific task and having three tasks makes it less likely to keep all tasks active in working memory. We expected to find reduced switch costs under increasing reward prospect and fastest repetition RTs and large switch costs when reward prospect remains high.^1^ This would provide further evidence for increased flexibility by increasing reward prospect and increased stability by remaining high reward prospect, and further support for sequential changes in reward expectation as an important modulator of meta-control.

## Experiments 1 and 2

We report methods and results for Experiments 1 and 2 together, because procedure and analyses in both experiments were mostly identical. The experiments only differed in terms of the specific tasks. In Experiment 1, we used a number and a letter task already used in previous studies (Fröber & Dreisbach, 2017) and added a new symbol task. A comparison between the three tasks (see *Supplemental Materials*) indicated that the symbol task was slightly more difficult in terms of an increased error rate. This might have been because only the number and letter task allowed for an intuitive compatible response mapping (Dehaene, Bossini, & Giraux, 1993; Gevers, Reynvoet, & Fias, 2003). Therefore, in Experiment 2, we used again the same symbol task but added two other tasks—a shape and a character task—without intuitive spatial compatibility. The between-tasks comparison (see *Supplemental Materials*) still indicated performance differences between the tasks in terms of RTs and error rates. However, with respect to our expectation to find a modulation of switch costs by the reward sequence, these between-task differences are uncritical, because we found reliable switch costs in all three tasks.

### Method

#### Participants

Sample size was determined with an a priori power analysis in G*Power 3.1.9.4 (Faul, Erdfelder, Lang, & Buchner, 2007). This analysis suggested a sample size of 29 participants to detect a medium-sized two-way interaction effect with a power of 95% and a significance level of 5%. This was rounded up to 30 participants. Two cohorts of undergraduate students from the University of Regensburg participated for course credit and the opportunity to win Amazon gift cards. We tested 30 participants in Experiment 1 (18-48 years, *M* = 24.9 years, *SD* = 7.24 years; all females) and another 30 participants in Experiment 2 (19-31 years, *M* = 21.57 years, *SD* = 2.51 years; 26 females). Participants gave written, informed consent before the experiment and were fully debriefed after completion in accordance with the ethical standards of the German Psychological Society and the 1964 Declaration of Helsinki. In each experiment, the best performing participant in terms of points earned during the reward phase was rewarded with a 15 € Amazon gift card, the second best with a 10 € Amazon gift card, and the third best with a 5 € Amazon gift card.

#### Apparatus, stimuli, and procedure

Both experiments were run on a PC with E-Prime 2.0 (Psychology Software Tools, Sharpsburg, PA). An LCD display (26 x 41 cm, 1440 x 900 px, 75 Hz) was used for stimulus presentation with an eye-monitor distance of approximately 60 cm. A QUERTZ keyboard was used for response collection with Y and M serving as left and right response key, respectively.

In both experiments, eight target stimuli per task were used. All target stimuli were presented 5% (approximately 2° visual angle) above the center of the screen in black on a white background. In Experiment 1, the numbers 125, 132, 139, 146, 167, 174, and 181 served as stimuli for the number task, the letters B, D, F, H, S, U, W, and Y served as stimuli for the letter task, and the symbols #, /, +, !, %, }, ~, and ? served as stimuli for the symbol task. Numbers and letters were presented in Calibri font, size 28, and symbols in Cambria font, size 28. Numbers had to be categorized as smaller or larger than 153, letters as closer to A or to Z in the alphabet, and symbols as to whether they contain straight lines only or also curved lines. For all participants, the left key was the correct response for numbers smaller than 153, letters closer to A, and symbols with straight lines only. This fixed response mapping was chosen in correspondence with the intuitive, spatial compatibility in the number and letter task (Dehaene et al., 1993; Gevers et al., 2003). In Experiment 2, the shapes ▲, , ■, ●, ♦, ♥, ♣, and ♠ served as stimuli for the shape task, and the characters , , , , Σ, Ω, ϕ, and Ψ served as stimuli for the character task. The third task was again the symbol task already used in Experiment 1. Shapes and characters were presented with a height of 50 px, and symbols in Cambria font, size 32 (resulting in roughly equal stimulus sizes across tasks). Shapes had to be categorized as basic geometric shapes or playing card symbols, and characters as Arabic or Greek letters. Due to the lack of an intuitive compatible mapping in the three tasks, response-to-category mapping to the left or right response key was counterbalanced across participants with playing card symbols, Arabic character, and straight lines always mapped to one response key, and geometric shapes, Greek character, and curved lines to the other response key. In both experiments, a central fixation dot (origin font, size 28) was used as reward cue. In low rewarded trials, the cue was presented in three different shades of gray (RGB values: 220, 220, 220; 169, 169, 169; 128, 128, 128), and in high rewarded trials in one of three colors (RGB values: 200, 124, 175; 235, 120, 95; 111, 156, 129). A low reward cue indicated the opportunity to win 1 point for an accurate response. A high reward cue indicated the opportunity to win 7 points for an accurate and fast response (faster than individually determined RT threshold, see below).

Both experiments consisted of three phases: practice, baseline, and reward. In the practice phase, participants were familiarized with all three tasks in short practice blocks of 16 trials each. Task order was counterbalanced across participants. This was followed by a short task switching practice block of 24 trials (all 8 stimuli of each task in random succession). After practice, participants progressed to a baseline block without reward manipulation of 192 trials. Trial order was pseudo-randomized with the exclusion of direct repetitions of target stimuli. The ratio of task repetitions to task switches was approximately 1:2. The nonreward baseline block was used to determine individual RT thresholds for the following reward phase. For each combination of task (1-3) and transition (repetition, switch) correct RTs were ordered from fast to slow and the fasted third was used as individual RT criterion. The reward phase comprised two blocks with 192 trials each, comprising half low reward and half high reward trials. Again trial order was pseudo-randomized: Direct repetitions of target stimuli were not allowed and each of the four reward sequences (remain low, increase, remain high, decrease) occurred about equally often.^2^ In addition, no direct repetitions of reward cue color was allowed, so that the physical appearance of the cue always changed even when reward magnitude remained the same (Logan & Schneider, 2006).

**Fig. 1:**
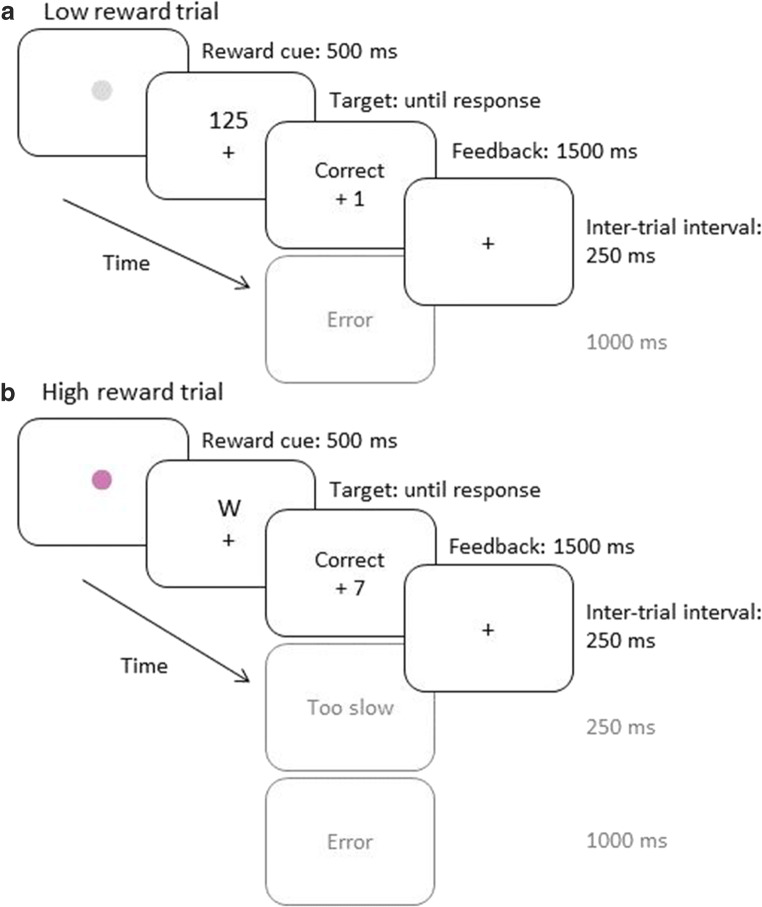
Procedure of a sample trial in Experiment 1 for the low reward (**A**) and high reward condition (**B**)

In the practice phase, each trial started with the presentation of a black fixation dot for 500 ms. The following target stimulus remained on screen until response. The response was followed by a feedback display for 1,000 ms (either “Correct!” or “Error!”). Each trial ended with an inter-trial interval of 250 ms after a correct response or 1,000 ms after an error. In the reward phase (Fig. 1), the fixation dot was replaced by one of the reward cues. In case of a low reward trial, the feedback now was either “Correct! +1 point” or “Error! No point”. In high reward trials, the feedback then read “Correct! +7 points” for correct responses faster than the individual RT threshold, “Too slow! No points” for correct but too slow responses, or “Error! No points” for erroneous responses.

#### Design

In both experiments, a 4 (reward sequence: remain low, increase, remain high, decrease) x 2 (task transition: repeat, switch) repeated-measures design was used. RTs (in ms) and error rates (in %) served as dependent variables.

### Results

#### Data preprocessing

We collapsed data across tasks since analyses of the baseline block without reward manipulation showed reliable switch costs for all three tasks (see *Supplemental Materials*).^3^ Practice trials, baseline trials, and the first trial of each reward block were excluded from all analyses. In addition, we excluded erroneous trials and trials following errors from RT analyses (Experiment 1: 15.76% of all data; Experiment 2: 22.95% of all data). Furthermore, RTs deviating more than ±3 standard deviations from individual cell means were excluded (Experiment 1: 0.39% of all data; Experiment 2: 0.89% of all data).

#### RTs

**Fig. 2:**
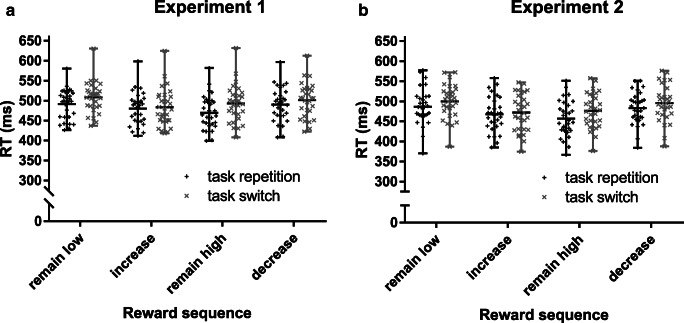
Mean RTs (in ms) and individual data points from Experiment 1 (**A**) and Experiment 2 (**B**) as a function of reward sequence (remain low, increase, remain high, decrease) and task transition (repeat, switch). Whiskers depict the range

A 4 (reward sequence) x 2 (task transition) repeated-measures ANOVA resulted in significant main effects of reward sequence, Experiment 1: *F*(3, 87) = 10.56, *p* < 0.001, η_p_^2^ = 0.267, Experiment 2: *F*(3, 87) = 18.60, *p* < 0.001, η_p_^2^ = 0.391, and task transition, Experiment 1: *F*(1, 29) = 29.36, *p* < 0.001, η_p_^2^ = 0.503, Experiment 2: *F*(1, 29) = 39.82, *p* < 0.001, η_p_^2^ = 0.579. These main effects were further qualified by a significant interaction of reward sequence x task transition (Fig. 2), Experiment 1: *F*(3, 78) = 5.51, *p* < 0.01, η_p_^2^ = 0.160, Experiment 2: *F*(3, 78) = 2.85, *p* < 0.05, η_p_^2^ = 0.089. In both experiments, participants were faster in high rewarded trials (increase, remain high) compared with low rewarded trials (remain low, decrease; *p*s < 0.01), while there was no significant difference within high reward (*p*s > 0.209) or low reward trials (*p*s > 0.107). More importantly with respect to our hypotheses, switch costs were modulated by reward sequence. Switch costs in increase trials were reduced to a nonsignificant difference in both experiments (Experiment 1: 3 ms, *p* = 0.471; Experiment 2: 4 ms, *p* = 0.259). To determine evidence for the null hypothesis, we conducted a Baysian analysis for this comparison resulting in moderate evidence for equal performance in repetition and switch trials (Experiment 1: BF_01_ = 4.02; Experiment 2: BF_01_ = 2.82). In contrast, typical switch costs ranging from 11 to 24 ms were found in all other reward sequences (*p*s < 0.05), while the largest switch costs were seen in remain high trials (Experiment 1: 24 ms, *p* < 0.001; Experiment 2: 19 ms, *p* < 0.001).

#### Error rates

**Fig. 3:**
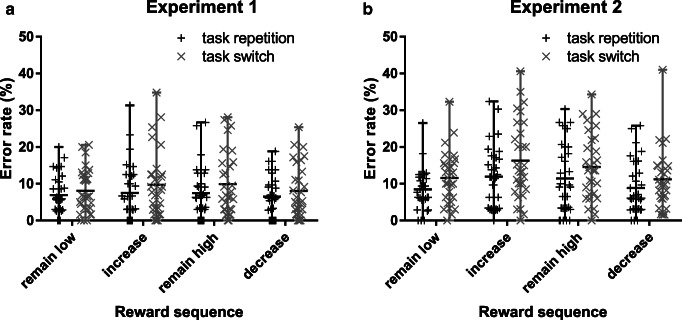
Mean error rates (in %) and individual data points from Experiment 1 (**A**) and Experiment 2 (**B**) as a function of reward sequence (remain low, increase, remain high, decrease) and task transition (repeat, switch). Whiskers depict the range

The same analysis on mean error rates resulted in a significant main effect of task transition, Experiment 1: *F*(1, 29) = 7.92, *p* < 0.01, η_p_^2^ = 0.215, Experiment 2: *F*(1, 29) = 16.85, *p* < 0.001, η_p_^2^ = 0.367. Participants showed typical switch costs (Experiment 1: 1.8%; Experiment 2: 3.2%) with more errors made in switch trials compared with repeat trials. The main effect of reward sequence was only significant in Experiment 2, *F*(3, 87) = 7.20, *p* < 0.001, η_p_^2^ = 0.199 (Experiment 1: *F* < 1, *p* = 0.409). Participants in Experiment 2 made more errors in high reward trials (increase, remain high) compared with low reward trials (remain low, decrease; *p*s < 0.05), whereas there was no significant difference within high (*p* = 0.117) or low reward trials (*p* = 0.981). In both experiments, the interaction of reward sequence x transition was not significant (*F*s < 1, *p*s > 0.760; Fig. 3).

#### RT analysis pooled across experiments

Both experiments resulted in a significant interaction of reward sequence x task transition in RTs. To investigate this interaction more closely, we collapsed data sets from both experiments to increase power for these post-hoc single comparisons. In addition, we report Bayes factors for all comparisons. Direct comparisons revealed that switch RTs were significantly faster in increase trials (478 ms) compared with remain low trials (504 ms, *p* < 0.001, BF_10_ = 573,640), remain high trials (485 ms, *p* < 0.001, BF_10_ = 53), and decrease trials (499 ms, *p* < 0.001, BF_10_ = 9465). In contrast, repetition RTs were significantly faster in remain high trials (463 ms) compared with remain low trials (589 ms, *p* < 0.001, BF_10_ = 347,763), increase trials (474 ms, *p* < .001, BF_10_ = 40), and decrease trials (487 ms, *p* < 0.001, BF_10_ = 15,058). Taken together, we found very strong evidence for fastest switch RTs in increase trials and fastest repetition RTs in remain high trials, whereas mean RTs collapsed across task transition did not differ significantly between increase and remain high trials (*p* = 0.225, BF_01_ = 3.46). In sum, both high reward conditions led to a comparable enhancement of performance, while task repetitions benefited especially by remaining high reward prospect and both task transitions benefited equally by increasing reward prospect.

### Discussion

Results from Experiments 1 and 2 demonstrate that both increasing and remaining high reward prospect motivated for equally enhanced performance, but they seemed to promote different modes of cognitive control. While remaining high reward prospect increased cognitive stability in terms of fastest switch RTs together with relatively large switch costs, increased reward prospect seemed to promote cognitive flexibility in terms of fastest switch RTs and negligible switch costs. No task cues were used in the task switching paradigm with three univalent tasks, so that advance preparation for a specific task would not make much sense. Furthermore, we assume that three tasks make it highly unlikely that participants would prepare for all tasks in response to an increase in reward. Instead we suggest that increasing reward prospect served as a meta-control signal to lower the updating threshold. This facilitates switching between tasks and leads to equal readiness to respond to any upcoming task be it a task switch or repetition.

As outlined in the *Introduction*, the sequential reward paradigm necessarily requires that low and high reward magnitudes are associated with different response requirements to assure a true performance-contingent reward manipulation. Previous studies addressed this confound of reward magnitude and response requirements in different ways: Shen and Chun conducted a control experiment with a speed instruction in both low and high reward trials (2011, Experiment 2). They replicated the key finding of smallest switch costs in reward increase trials, but also found some indications for task disengagement specifically in decrease trials (lower accuracy and highest RTs). We had a similar control experiment in our voluntary task switching version of the paradigm (Fröber & Dreisbach, 2016b, Experiment 3). The voluntary switch rate effect (higher switch rate in reward increase trials as compared to reward remain high trials) was the same as found with different response criteria for low and high reward trials, but in performance data the typical RT pattern was no longer present and the interaction was instead found in error rates. A different approach to deal with the issue of different RT thresholds per reward magnitude was used in Fröber et al. (2019). There, we used a voluntary task switching procedure with a double registration (Arrington & Logan, 2005). In this version of the paradigm, the task choice is registered prior to the target presentation and participants can take as much time as they need to make their decision. That is, only the response to the target is relevant for reward receipt, whereas the task choice RT is completely independent thereof. Nonetheless, the typical sequential reward effect on voluntary switch rates was replicated, strengthening the assumption that increased flexibility in reward increase trials and increased stability in reward remain high is not a mere consequence of changing response strategies.

To empirically address the different response strategies in low and high reward trials in this study, we decided to use the same approach as in Fröber et al. (2019). Therefore, we conducted an additional voluntary task switching experiment (Experiment 3) with three tasks this time using the double registration procedure (Arrington & Logan, 2005).

## Experiment 3

In Experiment 3, we used a voluntary task switching procedure with double registration and the same three tasks as in Experiment 1. In this paradigm the task choice is assessed in a separate response prior to the reward-relevant target response (Arrington & Logan, 2005; Fröber et al., 2019). Task choice was made without time pressure in both low and high reward trials and we measured the voluntary switch rate as an indicator of cognitive stability versus flexibility. That is, our stability-flexibility measure in this paradigm had the same response requirements for both reward magnitudes and was completely independent of the subsequent (reward-dependent) target response. Note, that we do not necessarily expect a replication of the RT interaction effect found in Experiments 1 and 2 due to the procedural differences in Experiment 3: With the double registration procedure, target RTs are measured only after the self-paced task-choice response. Thus, only a reduced impact of sequential changes in reward prospect on target RTs is expected. However, if the immediate reward history is indeed a modulator of meta-control, we should instead find a reduced voluntary switch rate in remain high trials indicating increased cognitive stability, and a higher voluntary switch rate in increase trials indicating high cognitive flexibility. As in Experiments 1 and 2, the low reward conditions are necessary to investigate sequential changes in reward prospect, but we refrain from a priori hypotheses regarding low reward trials (cf., Footnote 1).

### Method

#### Participants

Another 30 undergraduate students from the University of Regensburg participated in Experiment 3. Sample size was reduced to 28 participants (19-43 years, *M* = 23.82 years, *SD* = 5.94 years; 22 females) due to an E-Prime crash and exclusion of one participant with an extreme value in the voluntary switch rate (see *Supplemental Materials*). Again, the best performing participant in terms of points earned during the reward phase was rewarded with a 15 € Amazon gift card, the second best with a 10 € Amazon gift card, and the third best with a 5 € Amazon gift card.

#### Apparatus, stimuli, and procedure

Apparatus and stimuli were very similar to Experiment 1 with the following exceptions: Participants used the B, N, and M key with their right hand to choose the task, and the Y and X key to respond to the subsequent target stimulus. The same tasks as in Experiment 1 were used. </>, A/Z, and G/K^4^ served as choice prompts for the number, letter, and symbol task, respectively. Choice prompts appeared central and 10% left or right from central fixation (approximately 2.5° of visual angle) on the screen in Calibri font, size 28. The position of the choice prompts was counterbalanced across participants and participants chose the task with a spatially corresponding button press.

The same single task practice blocks as in Experiment 1 were followed by a voluntary task switching block of 16 trials to familiarize participants with the double registration procedure. To ensure that participants frequently switch between the tasks they were instructed to perform all three tasks about equally often, but in a random order (Arrington & Logan, 2004). As a visualization aid, they should imagine having a bowl with three balls, one for each task, and drawing one ball in each trial. Participants were discouraged from counting trial numbers per task or using repetitive sequences. The practice block was followed by a non-reward baseline block (174 trials) to determine individual RT thresholds like in Experiment 1. The following reward phase comprised two blocks with 192 trials each. Reward cues were pseudo-randomized like in Experiments 1 and 2.

**Fig. 4:**
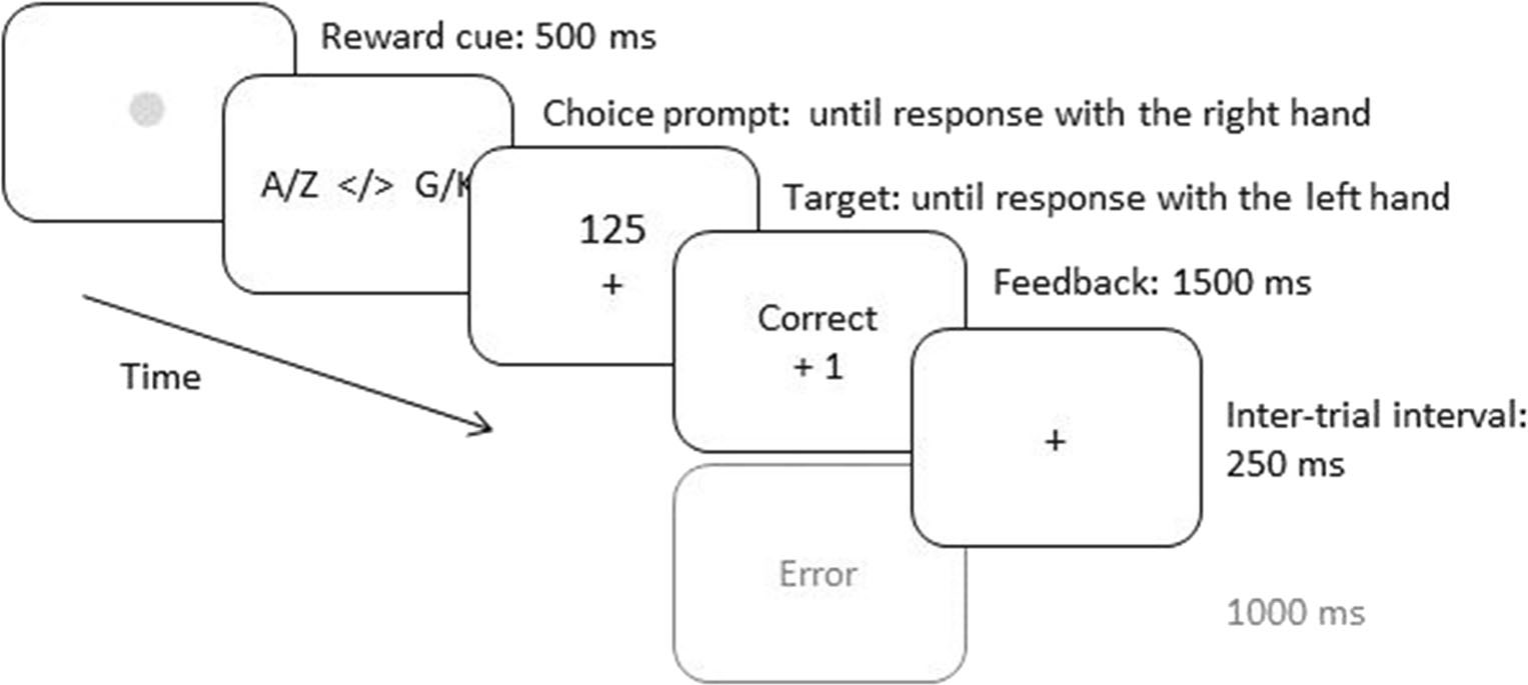
Procedure of a sample low reward trial with the double registration voluntary task switching procedure of Experiment 3. Note that both choice prompt and target display have no time limit for responding, but in a high reward trial the target RT needs to be faster than an individual RT threshold to actually receive a reward

The procedure of a single trial was like in Experiment 1 except for a choice prompt inserted between fixation dot/reward cue and target display (Fig. 4). The choice prompt was presented until the participant responded with no time limit or time pressure on task choice.

#### Design

Main dependent variable of Experiment 3 was the voluntary switch rate (in %) as a function of reward sequence (remain low, increase, remain high, decrease). For completeness, we also report analyses on choice RTs (CRT in ms; RTs to the choice prompt), target RTs (in ms), and target error rates (in %) with the additional repeated measures factor task transition (repeat, switch).

### Results

#### Data preprocessing

We collapsed data across tasks, because analyses of the baseline block without reward manipulation showed no significant effects including the factor task (see *Supplemental Materials*). Supplemental materials furthermore include some control analyses aimed at checking whether participants complied with the global instruction to perform all three tasks about equally often, but in a random order.

Practice trials, baseline trials, and the first trial of each reward block were excluded from all analyses. Analysis of the voluntary switch rate comprised all remaining trials, including errors to cover all attempts of deliberate switching (Arrington & Logan, 2004). We excluded erroneous trials and trials following errors from CRT and RT analyses (17.20% of all data). Furthermore, trials with CRTs or RTs deviating more than ±3 standard deviations from individual cell means were excluded (0.91% of all data).

#### Voluntary switch rate

**Fig. 5:**
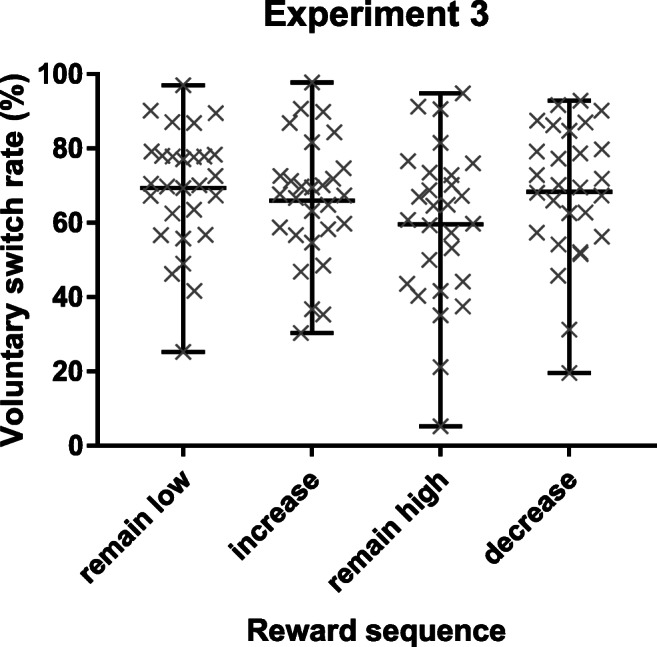
Mean voluntary switch rates (in %) and individual data points from Experiment 3 as a function of reward sequence (remain low, increase, remain high, decrease). Whiskers depict the range

A one-way repeated-measures ANOVA resulted in a significant main effect of reward sequence, *F*(3, 81) = 6.36, *p* < 0.001, η_p_^2^ = 0.191 (Fig. 5). We tested our hypothesis of the lowest voluntary switch rate in the remain high condition with planned one-tailed comparisons. The voluntary switch rate in remain high trials (59.64%) was significantly lower compared with remain low trials (69.34%; *p* < 0.001), increase trials (65.98 %; *p* < 0.05), and decrease trials (68.39%; *p* < 0.01). For the sake of completion, the voluntary switch rate in increase trials was significantly lower compared with remain low trials (*p* < 0.05) and did not differ significantly from decrease trials (*p* = 0.090). Bayes factors provided strong evidence for no difference between reward increase and low reward trials (increase vs. remain low: BF_01_ = 10.81; increase vs. decrease: BF_01_ = 14.98).

#### CRTs

A 4 (reward sequence) x 2 (task transition) repeated-measures ANOVA on CRTs resulted in no significant main effects or interaction (all *F*s < 2.17, all *p*s > 0.098).

#### RTs

**Fig. 6:**
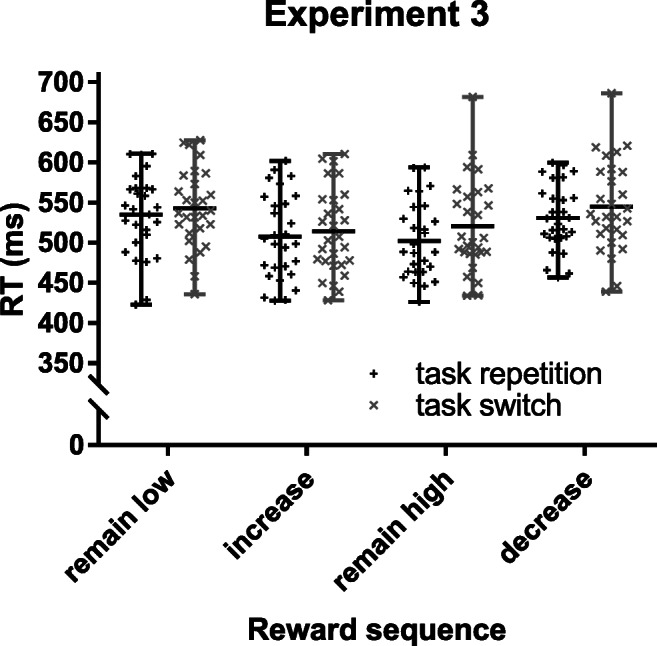
Mean RTs (in ms) and individual data points from Experiment 3 as a function of reward sequence (remain low, increase, remain high, decrease) and task transition (repetition, switch). Whiskers depict the range

Another 4 (reward sequence) x 2 (task transition) repeated-measures ANOVA on target RTs resulted in significant main effects of reward sequence, *F*(3, 81) = 12.29, *p* < 0.001, η_p_^2^ = 0.313, and task transition, *F*(1, 27) = 24.70, *p* < 0.001, η_p_^2^ = 0.478, but no significant interaction (*F* = 1.37, *p* = 0.259). Participants were faster in high reward trials (increase, remain high) as compared to low reward trials (remain low, decrease; *p*s < 0.01). No significant difference was found within low reward (*p* = 0.696, BF_01_ = 4.64) or high reward trials (*p* = 0.816, BF_01_ = 4.86). Participants showed typical, but rather small switch costs of 12 ms and, descriptively, the overall data pattern (Fig. 6) was similar to the RT pattern found in Experiments 1 and 2 with the smallest switch costs (7 ms) in reward increase trials and the largest switch costs (18 ms) in remain high trials.

#### Error rates

The same analysis on mean error rates resulted in no significant main effects or interaction (all *F*s < 1.71, all *p*s > 0.171).

### Discussion

Converging with results from Experiments 1 and 2 we again found evidence for increased flexibility or increased stability following the same high reward prospect, depending on the immediate reward history: participants switched tasks more often when reward prospect increased and switched tasks less often when reward prospect remained high. Importantly the task choice response had no time-restriction and was completely independent of the subsequent (reward-dependent) target response. These results confirm previous findings that task choice that is independent of reward receipt is still influenced by reward prospect in a systematic manner (Fröber et al., 2019).

Voluntary switch rates in low reward trials barely differed from those in increase trials which confirms previous findings that voluntary switching under low reward prospect is especially sensitive to the current task context. Note, that in a voluntary task switching paradigm with three tasks and a global instruction to choose all tasks about equally often but in a random order, voluntary switch rates should be close to 66.66% (see *Supplemental Materials*, analysis of the nonreward baseline block of Experiment 3). Thus we assume that the high voluntary switch rates in low reward conditions are (at least to some extent) a consequence of the flexibility required by the instruction, while the significant difference found between both high reward conditions still suggests the promotion of different cognitive control modes between increasing and remaining high reward prospect (for a more elaborate discussion see Fröber et al., 2019).

Target RTs showed a similar data pattern to RT results from Experiments 1 and 2. Switch costs were lowest in reward increase trials and highest in reward remain high trials, but the interaction effect was not significant. The lack of a significant interaction can probably be explained by the fact that target RTs in Experiment 3 were only measured after the reaction to the choice prompt. This necessarily extended the time between reward cue and response to the target stimulus and may therefore have dampened the sequential reward effect on RTs. Thus, it is not surprising that voluntary switch rates are the more sensitive measure with this procedure, given that the task choice response immediately follows the reward cue (Fröber et al., 2019). Taken together, prospect of the same high reward consistently promotes either cognitive stability or flexibility depending on the immediate reward history.

## General Discussion

The aim of the present study was to gather further evidence that increasing reward prospect increases cognitive flexibility. To this end, we used an uncued task switching paradigm with three univalent tasks together with a reward manipulation with two reward magnitudes in random succession. In the three experiments, participants showed enhanced performance in high reward trials (increase and remain high) compared with low reward trials (remain low, decrease). More importantly, RT switch costs were reduced to a nonsignificant difference (forced-choice task switching; Experiments 1 and 2) and voluntary switch rate was higher (voluntary task switching with double registration; Experiment 3) when reward prospect increased. In contrast, remaining high reward prospect specifically boosted task repetitions, slowed down task switches compared with reward increase trials (Experiments 1 and 2), and reduced the voluntary switch rate (Experiment 3). Because it is highly unlikely that participants used a strategy to prepare all three tasks in response to a cue announcing an increase in reward, this corroborates the assumption that sequential changes in reward expectation serve as a meta-control signal: increasing reward prospect biases the cognitive system towards higher cognitive flexibility and remaining high reward prospect towards cognitive stability (Dreisbach & Fröber, 2019).

The task switching paradigm with three univalent tasks and no task-specific cues does not allow advance preparation of a specific task. Thus, the non-existent switch costs under increased reward prospect cannot be explained by a mechanism that merely facilitates switching to the alternative task—as it was theoretically feasible in task switching procedures with two tasks only. Instead, the present results speak for a more generic form of cognitive flexibility when more reward than before can be expected. In fact, the prospect of a reward increase seems to have induced a state of equal readiness to perform any of the three potential tasks, be it a task repetition or a switch to one of the other tasks. Conversely, prospect of remaining high reward specifically facilitated task repetitions accompanied by pronounced switch costs. That is, remaining high reward prospect seems to stabilize the currently active task rule, resulting in costs when a different task has to be performed and a reduced willingness to voluntarily switch the task. Given the fact that advance task preparation is not a useful strategy in Experiments 1 and 2 and that task switches are more frequent than task repetitions in the current paradigm (and may therefore be expected), this repetition benefit is remarkable and provides more direct evidence for increased cognitive stability under remaining high reward prospect. Taken together, the present study confirms that prospect of the same high reward can either promote cognitive stability or flexibility depending on the immediate reward history, as has been suggested in previous (voluntary) task switching studies (Fröber et al., 2018; Fröber et al., 2019; Fröber et al., 2020; Fröber & Dreisbach, 2016b; Kleinsorge & Rinkenauer, 2012; Shen & Chun, 2011).

In the low reward prospect conditions (remain low and decrease trials), we found intermediate RT switch costs (Experiments 1 and 2) and higher voluntary switch rates compared with remaining high reward prospect (Experiment 3). In an exploratory analysis on RT switch costs from Experiments 1 and 2, including RT switch costs from the nonreward baseline block (see *Supplemental Materials*), switch costs were significantly smaller in all reward sequence conditions compared with baseline trials, except for remain high trials. Together with the finding that switch costs were reduced to a nonsignificant difference in the reward increase condition only, this suggests that increasing reward prospect leads to more cognitive flexibility and remaining high reward prospect to less cognitive flexibility than low reward prospect.^5^ Theoretically important, across both dependent variables (RTs and voluntary switch rates), we found converging evidence that the same high reward prospect has either a flexibility-increasing or a stability-increasing effect depending on the immediate reward history. Such within-reward magnitude differences were not found for the low reward conditions, which suggests that the sequential reward effect is not a mere consequence of changing versus unchanged reward expectation. Furthermore, the fact that we found a modulation of the voluntary switch rate by reward prospect in a double registration procedure without time pressure demonstrates once more that the sequential reward effect cannot be explained by different response requirements for low and high reward receipt (Fröber et al., 2019).

On a theoretical level, the present results corroborate the assumption that performance-contingent reward prospect is an important modulator of meta-control processes. Regarding the underlying mechanisms, we recently posited that increasing reward prospect might be a meta-control signal to lower the updating threshold in working memory (Dreisbach & Fröber, 2019). As a consequence, any information has equal chance of gaining access to working memory (Goschke & Bolte, 2014). The nonexistent difference between task repetitions and task switches and the relatively high voluntary switch rate perfectly fits with this assumption. In contrast, remaining high reward prospect seems to maintain or even increase the updating threshold, which stabilizes current representations in working memory and shields against competing information.^6^ This would perfectly explain the pronounced repetition benefit in RTs and the relatively low voluntary switch rate under remaining high reward prospect, even in a paradigm with predominant task switches.

As outlined in the introduction, a potential neurobiological implementation of the updating threshold might be a DA-mediated modulation of attractor states in prefrontal cortex (Durstewitz & Seamans, 2008; Rolls, 2010). Increasing reward prospect might promote the DA D2-receptor dominated state associated with shallow attractor states that facilitate switching between different working memory representations. Conversely, remaining high reward prospect might promote the DA D1-receptor dominated state associated with deep attractor states that are robust against interference and thus hard to switch away from. Related to this assumption, recent computational modelling work by Musslick and colleagues (Musslick, Bizyaeva, Agaron, Leonard, & Cohen, 2019; Musslick, Jang, Shvartsman, Shenhav, & Cohen, 2018) nicely demonstrates how variation in a single parameter can modulate the trade-off between cognitive stability and flexibility. In their model, it is not the depth of attractor states but the distance between attractors that is modulated by a gain factor. High gain means strong activation of one control attractor, but an increased distance to alternative control attractors. The opposite results from low gain, which facilitates switching between control attractors. This model has been successfully fitted to data from a task switching study with changing demands to cognitive flexibility (Musslick et al., 2019).

A complementary, not mutually exclusive explanation for the stability-flexibility balance can be found in the biologically based prefrontal cortex-basal ganglia-working memory model (PBWM; O'Reilly, 2006; O'Reilly & Frank, 2006). Therein, cognitive stability and flexibility are accomplished by a dynamic gating mechanism via NoGo and Go neurons located in the basal ganglia. Without a gating signal, NoGo neurons fire that inhibit thalamic neurons, thereby enabling the maintenance of bistable working memory representations in prefrontal cortex. When a gating signal is triggered, Go neurons are activated that open the gate to working memory by disinhibiting the thalamus, so that the bistable representations can be toggled to update working memory content. Triggering of a gating signal is assumed to depend on reward-related DA input to the basal ganglia: DA works excitatory on Go neurons via D1 receptors and inhibitory on NoGo neurons via D2 receptors. Thus, phasic DA bursts above tonic firing should increase Go firing and promote cognitive flexibility, whereas dips in DA below tonic firing should have the opposite effect. The PBWM model has also been successfully applied to the task switching paradigm (Herd et al., 2014).

There is neurobiological plausibility for both variants of computational models, direct modulation of stability versus flexibility via DA in prefrontal cortex (Durstewitz & Seamans, 2008) or indirect modulation via DA in the basal ganglia (O'Reilly & Frank, 2006). The first is assumed to have a broader, more global effect on working memory updating, while the letter enables selective updating of some working memory contents, whereas other information is kept maintained. While it is not entirely clear how both mechanisms (varying depths of attractor states in prefrontal cortex and dynamic gating via the basal ganglia) interact (O'Reilly, Herd, & Pauli, 2010), it seems reasonable to assume the following: If the updating threshold is low characterized by rather shallow attractor states in the prefrontral cortex, a rather weak gating signal might be sufficient to open the gate to working memory, whereas a high updating threshold characterized by rather deep attractor states might need a stronger gating signal to open the gate (Goschke & Bolte, 2014). This also would converge with behavioral findings from our lab, where we found that in a context of high stability (deep attractor states) only an increase in reward (strong gating signal) promotes cognitive flexibility, whereas in a context of high flexibility (shallow attractor states) any change in reward can further increase flexibility (Fröber et al., 2018).

While DA activity is associated with reward and especially unexpected changes in reward (Schultz, 2013), the neurotransmitter norepinephrine (NE) also might play an important role with respect to modulations of stability versus flexibility by reward. A newer version of the PBWM model (Hazy, Frank, & O'Reilly, 2007) implemented an exploration-exploitation mechanism as first introduced in the adaptive gain theory by Aston-Jones and Cohen (2005). Exploitation (as one facet of stability) refers to increased engagement in a given task and is supposed to be mediated by phasic NE activity in the locus coeruleus. Exploration (as one facet of flexibility) means facilitated engagement in alternative tasks, mediated by tonic NE activation. Importantly, the two NE activity modes are assumed to be driven by outcome utility. Under the assumption that outcome utility feeds future reward expectations, the predictions of the adaptive gain theory also may hold for reward prospect manipulations as applied in our experiments. For example, the exploitative mode is activated only as long as a given task is sufficiently rewarded. Thus, the stabilizing effect repeatedly observed under remaining high reward prospect also fits with the adaptive gain theory. And conversely, the prospect of an increase in reward may trigger a more explorative mode of control.

NE also might be involved in the sequential reward effect through learning mechanisms as explained in the adaptation by binding account of cognitive control (Verguts & Notebaert, 2009). An increase in reward prospect could elicit an increase in arousal (see Fröber et al., 2020, for recent pupillometric evidence for increased arousal by increasing reward prospect), which initially might lead to more impulsive behavior (Niv, Daw, Joel, & Dayan, 2007,7). The arousal then interacts with Hebbian learning by strengthening binding of active representations. Thus, on a subsequent high reward trial specifically task repetitions should be facilitated, which is exactly what we found in remain high trials.

Taken together, cognitive stability and flexibility are most likely mediated by a dynamic interplay of more than one neurotransmitter and future neuroscientific research should focus on clarifying the underlying neurobiological mechanisms from a systems-level perspective (Cohen et al., 2004), as is already done, for instance, in the latest version of the PBWM model (Hazy et al., 2007). And ideally, cognitive psychology should then use this neuroscientific insight to further inform and motivate behavioral paradigms that are suited to pinpoint the underlying cognitive mechanisms.

### Conclusions

By using an uncued task switching paradigm with three univalent tasks, the present study provides further evidence that increasing reward prospect promotes cognitive flexibility, whereas remaining high reward prospect promotes cognitive stability. The findings are suggestive of a mechanism of the sort that sequentially changing reward prospects modulate the updating threshold in working memory. More generally speaking, the present results endorse performance-contingent reward as a modulator of meta-control processes.

#### Open Practices Statement

Raw data for all experiments are available at https://epub.uni-regensburg.de/43661/.

## Electronic supplementary material

ESM 1 (DOCX 22 kb)

## Footnotes

1 We focus in the hypotheses on the high reward conditions, for which the largest motivational effect is expected and for which we have clear expectations based on previous research for both RT switch costs and voluntary switch rates. The low reward conditions are a necessity of the sequential reward manipulation, but so far it is less clear what to expect. With respect to RT switch costs, previous research (Shen & Chun, 2011) has found larger switch costs for remain low as compared to increase trials and intermediate switch costs for decrease trials. With respect to the voluntary switch rate, previous studies (Fröber et al., 2019; Fröber et al., 2020; Fröber & Dreisbach, 2016b) found that the voluntary switch rate was equally high in low reward trials (remain low, decrease) than in increase trials. Furthermore, voluntary switch rate in low reward trials seems to be very context-sensitive with very low voluntary switch rates found with a global context promoting cognitive stability (Fröber et al. 2018) or when switching to a more difficult task (Jurczyk et al., 2019). Because we have no experience with task switching paradigms with three tasks so far, we refrain from any a priori hypotheses regarding low reward trials.

2 An equal distribution of reward sequences (192 trials per block/4 sequences = 48 trials per sequence) was not possible, because the first trial in a block has no reward sequence. Above that, trial numbers per reward sequence were allowed to deviate ±3 trials from 48.

3 Only the error rate analysis of the baseline block from Experiment 1 did not results in a significant main effect of task transition. Importantly, task transition did also not interact with task in this analysis. Furthermore, we conducted an exploratory ANOVA of the reward phase including the factor task (which is not ideal due to small trial numbers per data cell especially for the less frequent repetition trial). We found significant main effects of transition, reward sequence, and task, but importantly no significant interaction of task with any other factor. Therefore, it seems noncritical to collapse across tasks to investigate the interaction of interest between reward sequence and task transition, thereby maximizing the number of data points per design cell.

4 Straight lines and curved line translate to “gerade Linien” and “kurvige Linien” in German and we used the first letters of these words as choice prompt for the symbol task.

5 For the remain high condition, this conclusion is supported by both dependent variables (RTs and voluntary switch rates). For the increase condition, only RT switch costs indicate less flexibility in low reward trials as compared to increase trials, whereas voluntary switch rates suggest comparable flexibility. Thus, performance in low reward trials seems to vary with respect to the dependent measure of interest.

6 Note that RT switch costs were descriptively largest in the nonreward baseline block (see *Supplemental Materials*). However, this comparison is hard to interpret, since any difference between baseline and reward phase is inherently confounded with practice.

7 These authors suggest in a similar manner that increases in reward cause more vigorous responding as a response to increased opportunity costs. Note, however, that they assume a DA-mediated mechanism.
