# Supplementary material for: How Sequentially Changing Reward Prospect Modulates Meta-control: Increasing Reward Prospect Promotes Cognitive Flexibility
Source: Cogn Affect Behav Neurosci. 2020 Sep 9;21(3):534–48. doi: 10.3758/s13415-020-00825-1 (PMC8208935; doi:10.3758/s13415-020-00825-1)
Supplement: Supplementary file 1 — (DOCX 22 kb) [file 13415_2020_825_MOESM1_ESM.docx]

Supplemental Material for:

**How sequentially changing reward prospect modulates meta-control: Increasing reward prospect promotes cognitive flexibility**

Kerstin Fröber & Gesine Dreisbach

1. **Reaction time (RT) and error rate analyses of the baseline block without reward manipulation from Experiments 1 and 2**

We conducted additional analyses on data from the baseline block without reward manipulation to compare performance between the three tasks. Furthermore, we wanted to check, if reliable switch costs emerge in the task switching paradigm with three univalent tasks used in the present study. The three tasks were presented in random order, so that a task switch was about twice as probable than a task repetition. Furthermore, no task cues were used, since the univalent target stimuli unequivocally determined the current task. Thus, participants could not prepare for a task repetition or switch prior to target onset. Criteria for data preprocessing were the same as for the analyses of the reward phase data reported in the main text.

**RTs**

Descriptive statistics from both experiments are shown in Table S1. A 3 (task: numbers, letters, symbols) x 2 (task transition: repeat, switch) repeated-measures ANOVA on RTs from Experiment 1 resulted in a significant main effect of task transition, *F*(1, 29) = 36.53, *p* < .001, η_p_^2^ = .557, which was further qualified by a significant interaction of task x task transition, *F*(2, 58) = 6.25, *p* < .01, η_p_^2^ = .177. Switch costs were significantly smaller in the number task (13 ms) as compared to the letter task (41 ms, *p* < .01) and the symbol task (43 ms, *p* < .01). Switch costs did not differ significantly between letter and symbol task (*p* = .849). The main effect of task did not prove reliable (*F* = 2.13, *p* = .128).

A 3 (task: shapes, characters, symbols) x 2 (task transition: repeat, switch) repeated-measures ANOVA on RTs from Experiment 2 resulted in significant main effects of task, *F*(2, 58) = 15.12, *p* < .001, η_p_^2^ = .343, and task transition, *F*(1, 29) = 29.03, *p* < .001, η_p_^2^ = .500. The interaction effect did not prove reliable (*F* = 2.26, *p* = .114). Participants were significantly slower in the symbol task (578 ms) as compared to the shape task (541 ms, *p* < .001) and the character task (541 ms, *p* < .001). Mean RTs did not differ between shape and character task (*p* = .923). Participants showed significant switch costs of 30 ms.

*Table S1*. Mean RTs (in ms; *SE* in parentheses) from Experiment 1 and 2 as a function of task and task transition.

|  | **Experiment 1** | | |
| --- | --- | --- | --- |
| **Task** | **Numbers** | **Letters** | **Symbols** |
| **Repeat** | 592 (20.69) | 588 (21.89) | 586 (17.21) |
| **Switch** | 632 (22.35) | 601 (21.27) | 629 (18.74) |
|  | **Experiment 2** | | |
| **Task** | **Shapes** | **Characters** | **Symbols** |
| **Repeat** | 522 (9.92) | 532 (8.88) | 561 (11.69) |
| **Switch** | 560 (11.29) | 550 (8.42) | 594 (18.85) |

**Error rates**

Descriptive statistics from both experiments are shown in Table S2. A 3 (task: numbers, letters, symbols) x 2 (task transition: repeat, switch) repeated-measures ANOVA on error rates from Experiment 1 resulted in a significant main effect of task, *F*(2, 58) = 6.10, *p* < .01, η_p_^2^ = .174. Participants made significantly more errors in the symbol task (3.95 %) as compared to the letter task (1.81 %, *p* < .001). The number task (2.42 %) did not differ significantly from either the symbol task (*p* = .063) or the letter task (*p* = .243). All other effects did not prove reliable (*F*s < 1.97, *p*s > .149).

A 3 (task: shapes, characters, symbols) x 2 (task transition: repeat, switch) repeated-measures ANOVA on error rates from Experiment 2 resulted in significant main effects of task, *F*(2, 58) = 9.08, *p* < .001, η_p_^2^ = .239, and task transition, *F*(1, 29) = 6.98, *p* < .05, η_p_^2^ = .194. Participants made significantly more errors in the symbol task (7.56 %) as compared to the shape task (5.11 %, *p* < .05) and the character task (3.52 %, *p* < .01), and significantly more errors in the shape task as compared to the character task (*p* < .05). Participants showed switch costs of 1.68 %.

*Table S2*. Mean error rates (in %; *SE* in parentheses) from Experiment 1 and 2 as a function of task and task transition.

|  | **Experiment 1** | | |
| --- | --- | --- | --- |
| **Task** | **Numbers** | **Letters** | **Symbols** |
| **Repeat** | 2.19 (0.73) | 1.75 (0.47) | 3.50 (0.95) |
| **Switch** | 1.44 (0.43) | 3.10 (0.58) | 4.40 (0.93) |
|  | **Experiment 2** | | |
| **Task** | **Shapes** | **Characters** | **Symbols** |
| **Repeat** | 4.70 (1.01) | 3.18 (0.83) | 5.80 (1.14) |
| **Switch** | 5.52 (1.04) | 3.85 (0.80) | 9.33 (1.41) |

**Summary and conclusion**

The control analyses showed some differences between tasks, but these seem noncritical for our main analyses of reward phase data. In all but one analysis, we found reliable switch costs in all three tasks. In the error rate analysis of Experiment 1 without a significant main effect of task transition, task transition did furthermore not interact with task. Thus, it seems justified to collapse data across tasks to investigate, if switch costs are modulated by reward sequence in the reward phase. The results from the baseline block suggest that RTs might be the more sensitive measure in the present paradigm.

1. **Exploratory comparison of RT switch costs from the non-reward baseline with switch costs in the four reward sequences for Experiments 1 and 2**

To see, if switching performance is affected by the reward context in a global manner, we conducted an exploratory repeated-measures ANOVA on RT switch costs (RT_switch_ – RT_repeat_) including the five levels baseline, remain low, increase, remain high, and decrease. Note that this analysis is necessarily confounded with practice, because the baseline block was always presented first. We report this exploratory analysis at the request of a reviewer. .

**Experiment 1**

The one-way repeated-measures ANOVA on switch costs in Experiment 1 resulted in a significant main effect, *F*(4, 116) = 7.82, *p* < .001, η_p_^2^ = .212. Contrast analyses revealed that baseline switch costs (33 ms) were significantly higher as compared to remain low trials (17 ms; *p* = .031), increase trials (3 ms; *p* < .001), and decrease trials (11 ms; *p* = .002). Switch costs did not differ significantly between the baseline and remain high trials (23 ms; *p* = .174).

**Experiment 2**

Another one-way repeated-measures ANOVA on switch costs in Experiment 2 resulted likewise in a significant main effect, *F*(4, 116) = 5.53, *p* < .001, η_p_^2^ = .160. Contrast analyses revealed that baseline switch costs (29 ms) were significantly higher as compared to remain low trials (14 ms; *p* = .015), increase trials (4 ms; *p* = .002), and decrease trials (12 ms; *p* = .005). Switch costs did not differ significantly between the baseline and remain high trials (23 ms; *p* = .078).

**Summary and Conclusion**

Results from both experiments show a consistent pattern with generally reduced switch costs in the reward phase, except for remain high trials. In remain high trials, switch costs were only descriptively reduced compared to the baseline. This might be a first hint that a reward context generally promotes cognitive flexibility, except for repeated high reward. Interpretation of these findings is however very limited due to the procedural confound. Practice effects might play a substantial role in the reduction of switch costs.

1. **Non-reward baseline block analyses for Experiment 3**

**Analyses testing the compliance of participants with the global task choice instruction**

Participants were instructed to choose all three tasks about equally often, but in a random order. The instruction to choose tasks randomly between the three tasks led to an expected voluntary switch rate of 66.66 %. We checked visually with a boxplot for extreme deviations from this value in the sample and identified one participant with an extremely low voluntary switch rate of 9.90 %. After exclusion of this participant, mean voluntary switch rate in the remaining sample was 71.37 %, which was remarkably high and even significantly higher than the expected switch rate (*p* < .05).

To test, if participants chose all three tasks about equally often we compared the absolute number of trials as a function of task (numbers, letters, symbols). The one-way repeated-measures ANOVA found no significant difference (*F* < 1, *p* = .821, BF_01_ = 7.53). Participants performed on average 63.0 trials of the number task, 64.4 trials of the letter task, and 63.6 trials of the symbol task.

**Analyses testing for differences between tasks**

Like in Experiments 1 and 2, we tested for task differences in target RTs and error rates. Descriptive statistics for Experiment 3 are shown in Table S3.

*Table S3*. Mean RTs (in ms; *SE* in parentheses) and error rates (in %; *SE* in parentheses) from Experiment 3 as a function of task and task transition.

| **Task** | **Numbers** | **Letters** | **Symbols** |
| --- | --- | --- | --- |
|  | **RTs** | | |
| **Repeat** | 613 (16.76) | 613 (15.36) | 606 (11.26) |
| **Switch** | 636 (17.67) | 641 (19.45) | 660 (17.64) |
|  | **Error rates** | | |
| **Repeat** | 3.06 (0.93) | 3.84 (1.23) | 3.75 (1.07) |
| **Switch** | 2.94 (0.73) | 4.72 (0.86) | 8.09 (1.44) |

***RTs***

A 3 (task: numbers, letters, symbols) x 2 (task transition: repeat, switch) repeated-measures ANOVA on RTs from Experiment 3 resulted in a significant main effect of task transition, *F*(1, 27) = 18.90, *p* < .001, η_p_^2^ = .412, which was further qualified by a significant interaction of task x task transition, *F*(2, 54) = 3.53, *p* < .05, η_p_^2^ = .116. Switch costs were significantly larger in the symbol task (54 ms) as compared to the letter task (28 ms, *p* < .05) and the number task (23 ms, *p* < .05). Switch costs did not differ significantly between letter and symbol task (*p* = .723). The main effect of task was not significant (*F* < 1, *p* = .783).

***Error rates***

The same analysis on error rates from Experiment 3 resulted in significant main effects of task, *F*(2, 54) = 3.99, *p* < .05, η_p_^2^ = .129, and task transition, *F*(1, 27) = 4.43, *p* < .05, η_p_^2^ = .141, which were further qualified by a significant interaction of task x task transition, *F*(2, 54) = 4.19, *p* < .05, η_p_^2^ = .134. Participants made significantly more errors in the symbol task (3.95 %) as compared to the letter task (1.81 %, *p* < .001). Switch costs were significantly larger in the symbol task (4.34 %) as compared to the number task (-0.12 ms, *p* < .01) and marginal significantly larger as compared to the letter task (0.88 %, *p* = .055). Switch costs did not differ significantly between letter and symbol task (*p* = .534).

**Summary and conclusion**

The control analyses on task choice showed that participants as a group showed no indication of a repetition bias and good compliance with the global task choice instructions. Target RT and error rate analyses showed like in Experiments 1 and 2 some differences between tasks, but these again seem noncritical for our main analyses of reward phase data. In all but one condition, we found reliable switch costs in all three tasks. Thus, it seems again justified to collapse data across tasks.
